# Factors Associated with Unplanned Dialysis Starts in Patients followed by Nephrologists: A Retropective Cohort Study

**DOI:** 10.1371/journal.pone.0130080

**Published:** 2015-06-05

**Authors:** Pierre Antoine Brown, Ayub Akbari, Amber O. Molnar, Shaurya Taran, Janice Bissonnette, Manish Sood, Swapnil Hiremath

**Affiliations:** 1 Division of Nephrology, Department of Medicine, University of Ottawa, Ottawa, Ontario, Canada; 2 Kidney Research Centre, Ottawa Hospital Research Institute, Ottawa, Ontario, Canada; 3 Clinical Epidemiology Program, Ottawa Hospital Research Institute, Ottawa, Ontario, Canada; 4 University of Ottawa, Ottawa, Ontario, Canada; Rouen University Hospital, FRANCE

## Abstract

The number of patients starting dialysis is increasing world wide. Unplanned dialysis starts (patients urgently starting dialysis in hospital) is associated with increased costs and high morbidity and mortality. Risk factors for starting dialysis urgently in hospital have not been well studied. The primary objective of this study was to identify risk factors for unplanned dialysis starts in patients followed in a multidisciplinary chronic kidney disease (CKD) clinic. We performed a retrospective cohort study of 649 advanced CKD patients followed in a multidisciplinary CKD clinic at a tertiary care hospital from January 01, 2010 to April 30, 2013. Patients were classified as unplanned start (in hospital) or elective start. Multivariable logistic regression was used to identify variables associated with unplanned dialysis initiation. 184 patients (28.4%) initiated dialysis, of which 76 patients (41.3%) initiated dialysis in an unplanned fashion and 108 (58.7%) starting electively. Unplanned start patients were more likely to have diabetes (68.4% versus 51.9%; p = 0.04), CAD (42.1% versus 24.1%; p = 0.02), congestive heart failure (36.8% versus 17.6%; p = 0.01), and were less likely to receive modality education (64.5% vs 89.8%; p < 0.01) or be assessed by a surgeon for access creation (40.8% vesrus78.7% p < 0.01). On multivariable analysis, higher body mass index (OR 1.07, 95% CI 1.02, 1.13), and a history of congestive heart failure (OR 2.41, 95% CI 1.09, 5.41) were independently associated with an unplanned start. Unplanned dialysis initiation is common among advanced CKD patients, even if they are followed in a multidisciplinary chronic kidney disease clinic. Timely education and access creation in patients at risk may lead to lower costs and less morbidity and mortality.

## Introduction

Some patients initiate permanent dialysis as inpatients in an unplanned fashion. Recent studies have linked this type of dialysis start to poorer patient outcomes over both the short- and long-term.[1] Patients who initiate long-term dialysis as inpatients are less likely to be on peritoneal dialysis. Moreover, they are more likely to start dialysis with a central venous catheter rather than a fistula.[1–4] Such factors contribute to the economic burden of chronic kidney disease (CKD), with costs that are already very high and climbing.[5, 6] For some patients, unplanned dialysis initiation may be unavoidable, due to either sudden-onset illness or acute unforeseeable renal decline.[7] Several studies have identified late referral to a nephrologist as a major cause for unplanned starts to dialysis,[8–10] though early referral by itself does not ensure optimal dialysis start.[11]

Many patients with advanced CKD are now managed in specialized multi-disciplinary clinics (MDCs), where the primary goal is to transition patients smoothly onto dialysis. The efficacy of MDCs has been well studied, with reports showing that they decrease mortality and hospitalizations in CKD patients, [12] measurably improve patient performance and considerably reduce health care costs.[12] As a result, MDCs are now increasingly prevalent as part of large CKD programs. However, despite the many benefits that MDCs offer patients, a significant proportion of patients followed in such clinics still initiate dialysis in an unplanned fashion during a hospital admission, rather than electively as outpatients.[4, 11]

Characterizing factors that might predict the need for unplanned dialysis in patients followed in MDCs is of particular importance, since it may allow CKD programs to reduce the incidence of unplanned dialysis initiations. This would save costs, could lead to less morbidity and mortality, and better access planning. With this objective, we undertook the present study to identify risk factors that could help predict the need for unplanned dialysis initiation among patients with advanced CKD. Secondary objectives included examining the differences between patients starting dialysis urgently versus patients starting dialysis electively.

## Subjects and Methods

### Setting and Patients

This study was conducted at the Ottawa Hospital (TOH), a 1,150 bed academic tertiary care center serving a population of approximately 1.2 million habitants located in Ontario, Canada. TOH’s CKD program is the sole CKD program in the catchment area. The study population consisted of patients who were being followed for advanced CKD at the MDCs, a specialty multi-disciplinary care clinic in the CKD program for patients approaching end-stage renal disease (ESRD). Patients are referred to the clinic by their primary nephrologists in anticipation of ESRD. Timing of transfer is at the discretion of the primary nephrologist, though referrals are suggested when the eGFR is < 25mL/min/1.73 m2 (calculated using the MDRD formula). The clinic is staffed by nurses, social workers, dieticians, pharmacists and physicians. Patients are seen in the MDC regularly, sometimes as often as every two weeks and at a minimum twice a year, though the exact interval is left to the discretion of the clinician. At each visit, patients are seen by the nurse, dietician and physician, with the pharmacist and social worker available as needed.

### Data Collection

Data is collected at the end of each routine clinic visit and entered into a database of our MDC clinic. The accuracy of the data in the database is verified every six months by auditing 5% of entries and accuracy has been > 95% since inception. Inclusion of comorbidities in the clinical database used for our manuscript is based on the referring nephrologist completing a mandatory detailed co-morbidity questionnaire at the time of referral. pecifically for congestive heart failure (CHF), a diagnosis was made by echocardiographic findings or on clinical grounds including hospitalization for acute decompensation prior to first MDC clinic visit. Dyslipidemia was defined as abnormal lipid profile or if the patient was on a lipid lowering medication. Cerebrovascular event was defined by documentation based on a neurologists note or based on nephrologists clinical judgement or imaging studies of brain indicating cerebrovascular event. Data on all patients followed in the clinic since the inception of the database to the last follow up date available at the time of data analysis (January 01, 2010 to April 30, 2013) were retrieved from the database for this study. Data on baseline characteristics, the presence of relevant co-morbidities, and laboratory information was abstracted from the database.

For each patient that initiated dialysis, the type of dialysis start (unplanned versus elective) was ascertained by a comprehensive chart review of the TOH electronic health record systems (vOacis; Telus Healthcare & Nephrocare; Fresenius Medical Care, Bad Homburg, Germany) by two authors (ST & PAB) independently. Patients were defined as having an unplanned start only if they started dialysis as inpatients in the hospital. At our institution, tunneled dialysis catheters are inserted by interventional radiologists. Appointments for such procedures can normally be obtained within 24–48 hours of referral and as such, there is no need to electively admit patients to initiate dialysis, even if a permanent access is not present at the time of need for dialysis. Hence the only patients who get admitted and start dialysis as an inpatient are those who have an acute indication.

The clinical indications for dialysis initiation were defined as: hyperkalemia (serum K of >5.5 mEq/L resistant to medical therapy), volume overload (pulmonary radiography documenting evidence of interstitial pulmonary edema), uremia (unequivocal physician note detailing uremic pericarditis, asterixis, increasing lethargy, metallic taste in the mouth, and/or nausea/vomiting), and other/unknown. If multiple reasons were equally likely to explain the need for dialysis, then that patient was assigned to all of the appropriate categories. Classification conflicts were resolved by review and consensus of all authors. The Ottawa Hospital Research Ethics Board reviewed and approved this study. Patient records were anonymized and de-identified prior to analysis.

### Statistics

Continuous variables are reported as mean (SD) or median (IQR) and categorical variables are reported as numbers (%). Univariate comparisons between the elective and unplanned starts groups were performed using Student’s t-tests and Chi-square tests, where appropriate. Based on these preliminary comparisons, all variables identified as significant (at p <0.1) were then entered into a logistic regression in order to identify factors that were associated with patients initiating unplanned dialysis. All analyses were performed using *SAS* (version 9.3). A p value of < 0.05 was considered significant.

## Results

A total of 649 patients were included in the study. The baseline characteristics are presented in Table 1. Median follow up time was 1.01 (IQR 1.2) years after referral to the clinic.

**Table 1:** Baseline Characterisitics.

Characteristic	Number of patients = 649
Age, years (SD)	66.2 (15.5)
BMI, kg/m^2^ (SD)	29.9 (7.1)
Gender, female n (%)	381 (58.7%)
**Race**	
Caucasian, n (%)	474 (73.0%)
African, n (%)	38 (5.9%)
Other, n (%)	137 (21.1%)
**Cause of CKD**	
Diabetes, n (%)	294 (45.3%)
Ischemic nephropathy, n (%)	128 (19.7%)
Glomerulonephritis, n (%)	90 (13.9%)
Polycystic kidney disease, n (%)	42 (6.5%)
Other, n (%)	95 (14.6%)
**Co-morbidities**	
Diabetes, n (%)	365 (56.4%)
Coronary artery disease, n (%)	196 (30.4%)
Hypertension, n (%)	592 (91.9%)
Congestive heart failure, n (%)	171 (26.5%)
Cerebrovascular accident, n (%)	73 (11.4%)
Dyslipidemia, n (%)	445 (69.3%)
Creatinine, μmol/L (SD)	239 (80)
eGFR, ml/min/1.73m^2^ (SD)	17.7 (5.9)
Albumin to creatinine ratio, mg/mmol (SD)	222 (321)
Hemoglobin, g/L, mean (SD)	111.5 (15.6)
Urea, mmol/L, mean (SD)	20.9 (7.3)
Albumin, g/L, mean (SD)	34.6 (4.9)

One hundred and eighty four (28.4%) patients initiated renal replacement therapy during the period of the study, 70 (10.8%) patients died, and 41(6.3%) patients were transferred out of MDC including 18 (2.8%) who had a pre-emptive renal transplant. Of the 184 who initiated dialysis, 76 patients (41.3%) initiated dialysis in an unplanned fashion and 108 (58.7%) began dialysis electively. In the unplanned start group, 75 (98.7%) were initiated on hemodialysis (HD), with only one patient initiating unplanned peritoneal dialysis (PD). In the elective start group, 65 (60.2%) started on HD and 43 (39.8%) started PD. Both patient groups were quite similar in age at referral to the MDC, age at the start of dialysis, race, gender, cause for ESRD (with the exception of a higher proportion of polycystic kidney disease patients in the elective start group), and length of follow-up time (minimum follow up in both groups one week and mximum follow up in elective group 138 weeks and unplanned group 121 weeks) (Table 2). The three most common co-morbidities for both groups of patients were hypertension, dyslipidemia, and diabetes. However, there were significant differences between the groups, with patients who initiated unplanned dialysis having a higher rate of coronary artery disease (CAD) (42.1% vs 24.1%; p = 0.02), diabetes (68.4% vs 51.9%; p = 0.04) and CHF (36.8% vs. 17.6%; p = 0.01) compared to patients who initiated dialysis electively. Similarly, differences between groups were found for eGFR at referral, and prior to dialysis initiation; these were significantly higher in the unplanned start group. Proteinuria documented by albumin to creatinine ratio did not differ significantly between the two groups.

**Table 2:** Characteristics of patients starting dialysis.

	Elective start n = 108	Unplanned start n = 76	Difference (95% CI)	P value
Age, years, mean (SD)	61.1 (15.8)	64.9 (15.5)	3.8 (-0.83, 8.43)	0.11
Gender, Female, n (%)	69 (63.9)	41 (54)	9.9% (-5.2, 24.8)	0.23
BMI, kg/m^2^, mean (SD)	28.2 (5.9)	31.2 (7.3)	3 (1.08, 4.92)	<0.01
**Race**				
Caucasian, n (%)	76 (70.4)	51 (67.1)	3.3% (-10.8, 17.7)	0.75
African, n (%)	10 (9.3)	5 (6.6)	2.7% (-6.7, 11.1)	0.7
Other, n (%)	22 (20.4)	25 (32.9)	12.5% (-1.1, 26.2)	0.08
**Cause of CKD**				
Diabetes, n (%)	47 (43.5)	45 (59.2)	15.7% (0.3, 30.4)	0.05
Ischemic nephropathy, n (%)	17 (15.7)	11 (14.5)	1.2% (-10.6, 12.0)	0.99
Glomerulonephritis, n (%)	24 (22.2)	10 (13.2)	9% (-3.2, 20.2)	0.18
Polycystic kidney disease, n (%)	13 (12)	1 (1.3)	10.7% (2.7, 18.5)	0.02
Other, n (%)	7 (6.5)	9 (11.8)	5.3% (-3.7, 15.5)	0.32
**Co-morbidities**				
Diabetes, n (%)	56 (51.9)	52 (68.4)	16.5% (1.3, 30.7)	0.04
Coronary artery disease, n (%)	26 (24.1)	32 (42.1)	18% (3.5, 32.1)	0.02
Hypertension, n (%)	106 (98.2)	71 (93.4)	4.8% (-1.6, 13.1)	0.2
Congestive heart failure, n (%)	19 (17.6)	28 (36.8)	19.2% (5.5, 32.8)	0.01
Cerebrovascular accident, n (%)	9 (8.4)	9 (11.8)	3.4% (-5.9 to 13.9)	0.61
Dyslipidemia, n (%)	65 (60.2)	51 (47.2)	13% (-2.4, 27.8)	0.11
Time followed, (Days)	281 (227)	350 (264)	76 (8, 144)	0.07
Creatinine at first visit, μmol/L, mean (SD)	399 (126)	341 (101)	-58 (-24, -92)	<0.01
Number of Visits	3.6 (2.1)	2.7 (1.8)	-0.9 (-1.5, 0.3)	<0.01
On renin angiotensin system blocker, n (%)	51 (47.2)	33 (43.4)	3.8% (-11.5, 18.8)	0.72
eGFR at first visit, ml/min/1.73m^2^ (SD)	13.7 (4.7)	15.5 (5)	1.8 (0.4, 3.2)	0.01
Creatinine at start of dialysis, μmol/L, mean (SD)	587 (157)	391 (117)	-196 (-154, -238)	<0.01
eGFR at start of dialysis, ml/min/1.73m^2^, mean (SD)	8.5 (2.3)	13 (3.7)	4.5 (3.6, 5.4)	< 0.01
Albumin to creatinine ratio, mg/mmol, mean (SD)	316 (284)	352 (330)	36 (-54, 126)	0.43
Hemoglobin, g/L, mean (SD)	109 (14.2)	106 (17.1)	-3 (-7.6, 1.6)	0.20
Urea, mmol/L, mean (SD)	22.8 (6.9)	23.3 (8.7)	0.5 (-1.8, 2.8)	0.70
Albumin, g/L, mean (SD)	33.6 (4.8)	31.5 (5.8)	-2.1 (-3.7, -0.6)	<0.01

Patients in the unplanned start group were much less likely to have received formal education about the different options for renal replacement therapies before they started dialysis (52.6% vs 14%; p<0.01), and were less likely to have been evaluated by a surgeon for creation of a dialysis access (59.2% vs 21.3%, p<0.01). Finally, patients starting in an unplanned fashion were also less likely to have a permanent access present when dialysis was initiated (10.5% vs 52.8%; p<0.01; Table 3).

**Table 3:** Education and Access status.

	Elective start n = 108	Unplanned start n = 76	Difference (95% CI)	P value
Education about Modalities of RRT, n (%)	97 (89.8)	49 (64.5)	25.3% (12.4, 38.1)	<0.01
Seen in access clinic, n (%)	85 (78.7)	31 (40.8)	37.9% (23.0, 51.2)	< 0.01
Fistula or graft, n (%)	14 (13)	7 (9.2)	3.8% (-6.7, 13.3)	0.6
Central venous catheter, n (%)	51 (47.2)	68 (89.5)	42.3% (28.9, 53.6)	<0.01
Peritoneal dialysis catheter, n (%)	43 (39.8)	1 (1.3)	38.5% (27.6, 48.5)	<0.01
Permanent access, n (%)	57 (52.8)	8 (10.5)	42.3% (28.9, 53.62)	<0.01

RRT, Renal replacement therapy

Reasons for initiating dialysis were different between groups. yperkalemia was more likely to have contributed to unplanned dialysis starts than elective dialysis starts (18.4% vs 3.7%; p = 0.01). The same association was found for volume overload (51.3% vs 21.3%; p = <0.01). Uremia was much less likely in the unplanned start group (32.9% vs 59.35%; p<0.01), but was the most common indication in the elective start group. Of note, 33 (30.6%) patients in the elective start group and 15 (19.7%) in the unplanned start group had more than one reason to initiate RRT. Each reason was analyzed independently.

In the multivariable analysis using logistic regression (Table 4), a higher BMI (OR 1.07 per unit change, 95% CI 1.02, 1.13; p = 0.006) and history of CHF (OR 2.41, 95% CI 1.09, 5.41; p = 0.04) were associated with unplanned start. Conversely, the presence of hypertension (OR 0.08, 95% CI 0.004, 0.51; p = 0.02) was associated with an elective start.

**Table 4:** Multivariable analysis.

	Adjusted Odds Ratio	95% CI	p value
Body Mass Index (per unit kg/m^2^ increase)	1.07	1.01, 1.13	0.03
Diabetes	1.10	0.51, 2.41	0.77
Coronary Artery Disease	1.54	0.72, 3.29	0.16
Congestive Heart Failure	2.41	1.09, 5.41	0.04
Polycystic Kidney Disease	0.23	0.01, 1.36	0.19
Hypertension	0.08	0.004, 0.53	0.02
Baseline GFR (per unit mL/min/1.73m^2^ increase)	1.05	0.98, 1.13	0.18
Albumin (per unit g/L increase)	1.02	0.94, 1.10	0.67

## Discussion

Unplanned dialysis is a common occurrence among CKD patients.[13, 14] In the primary care population, late referral to a nephrologist has been identified as a robust predictor of unplanned dialysis.[15, 16] However, factors associated with unplanned start of dialysis in patients who are already followed by nephrologists in a MDC clinic are not clearly defined. Our data suggests that higher BMI and a history of CHF are associated with unplanned dialysis starts in this population.

There is limited existing data on unplanned dialysis initiation in patients followed in the MDC clinics.[1] Our results add to the existing data and highlight several important findings. First, the rate (40%) of unplanned dialysis initiation at our center and that PD is strongly associated with an optimal start is in keeping with previously published data.[4, 11, 14] However, this can be of little comfort, since the unplanned start rate is unacceptably high. Our findings and others[14] suggest that, even with early referral and regular follow-up, unplanned dialysis starts still occur frequently. Therefore, to improve ESRD patient outcomes, it is necessary to move beyond the focus on early referral to a nephrologist and the simple existence of CKD MDCs and identify further factors contributing to unplanned starts and ultimately reduce the incidence of these events.

Data in this regard is limited and most studies have examined an outcome of suboptimal or unplanned dialysis starts defined as either patients starting dialysis during a hospitalization or starting as an outpatient with a catheter. Buck et al.[1] compared patients followed by a nephrologist for ≥ 4 months to those followed in MDC clinics and found that MDC patients were less likely to be initiated on dialysis urgently and that there was no difference in patient characteristics between unplanned and elective starts. Their results may differ from ours due to the limited sample size (n = 69 patients followed in the MDC). Another contributor could be the fact that the definition of an unplanned dialysis differs between our study and theirs. uck et al[1] defined unplanned dialysis starts as patients either starting hemodialysis with a catheter in an outpatient setting, or starting dialysis in hospital, whereas we restricted unplanned starts to dialysis initiation occurring during a hospitalization. We choose this definition because a majority of patients have a catheter at their first outpatient dialysis[17] and comparing patients who initiate dialysis with a catheter versus a fistula is inherently biased[18]. At our centre, a subgroup of patients have a planned initiation with a catheter. We feel that it would be inappropriate to include such patients in the unplanned group. A planned initiation with a catheter may occur for a number of reasons. Certain CKD patients, after being assessed by the vascular surgeon, are found to have very poor veins therefore requiring vein transposition for creation of a fistula. Others may have previously had a fistula that failed or a perceived higher risk of failure once created.[19, 20] For these reasons, fistula creation may be delayed until after dialysis initiation. This approach helps to minimize exposure of patients to repeated or unnecesarry procedures. Finally, another subgroup of patients who start dialysis electively with a catheter as an outpatient are elderly patients who have selected hemodialysis in whom a prior assessment of early access creation was deemed to be of uncertain benefit. In all of these situations, although patients are starting dialysis with a catheter, we would consider that the patient has had an optimal start; he or she started the dialysis modality of their choice in a planned fashion. Conversely, at our centre patients starting dialysis during a hospitalization are almost universally unplanned. Outpatient permanent catheter insertions (performed by interventional radiology) are relatively easy to obtain on short notice, and hospital admissions are avoided unless absolutely necessary due to the increased cost, limited availability of hospital beds, increased patient inconvenience, and potential increased morbidity. Our centre’s experience that few patients require in hospital initiation of dialysis due to a lack of outpatient resources is supported by a previously published study.[14]

Our study also demonstrates that patients starting unplanned dialysis were less likely to have received education about all modalities of renal replacement therapy and an attempt at access creation, which is similar to the findings of prior studies.[1, 2] Since there is no reason to believe that there was a difference in the level of care between unplanned and elective starters, it is likely that certain unplanned start patients sustained an acute, perhaps unforeseeable, decline in renal function, which led to dialysis initiation. Although we did not measure the trajectory of kidney function decline prior to dialysis initiation, a previously published study found that a large majority (30%) of unplanned start patients experience an acute decline in renal function. lower rate of access creation or dialysis education could therefore be associated with unplanned starts due to a greater number of patients in the unplanned group experiencing an unforeseeable acute decline in renal function as opposed to these factors contributing to the cause of unplanned starts. Research has indeed shown that not all CKD patients have a steady decline in kidney function, but that some patients experience a steep decline, or even a sudden progression to kidney failure.[21] The hypothesis of many unplanned start patients experiencing a sudden decline in kidney function is supported by the fact that proteinuria, a well-known predictor of renal function decline,[22] did not differ between groups in our study, and eGFR at dialysis initiation was higher for patients who had an unplanned start, suggestive of an acute indication to initiate dialysis rather than a slow progression of uremic symptoms. In fact, patients in the unplanned start group were much more likely to necessitate dialysis initiation for hyperkalemia or volume overload rather than uremia.

Most importantly, we report patient characteristics associated with unplanned dialysis starts. Patients with a higher BMI and a history of congestive heart failure appear to be much more likely to require an unplanned dialysis start. A higher BMI is associated with diabetes and atherosclerosis, both of which are cardiovascular risk factors; therefore, obese patients may acutely decompensate due to vascular events necessitating unplanned dialysis. Surprisingly, hypertension seemed protective, a finding perhaps explained by the fact that most patients with significant systolic heart failure are chronically normo- or even hypotensive. Although the risk factors we report may not cause an acute worsening of kidney function, we speculate that they could represent a particular patient profile at higher risk of such catastrophic events. To support our conclusion, it was much more likely that the reason for dialysis initiation in the unplanned start group was pulmonary edema or hyperkalemia, rather than uremia. Indeed, it has been recently reported that congestive heart failure is not only common in CKD patients, but that it also increases risk of subsequent need for RRT.[23]

Though our findings might be perceived as not entirely unexpected, they are clinically very relevant. Knowing that these factors may heighten the risk of unplanned dialysis start could help nephrologists flag high-risk patients. This might lead nephrologists to plan more frequent visits to the MDC since it has been shown that the cumulative amount of care received in a CKD MDC, rather than simply just time spent followed in a MDC appears to influence the likelihood of having an optimal dialysis start.[11] Patients at increased risk of an unplanned start would also likely benefit from early education efforts, which would better support their decision-making around end-stage renal disease options. Finally, these patients may also be referred for earlier attempts at dialysis access creation, thus increasing the number of patients who start dialysis with optimal access—which to date remains suboptimal in patients starting RRT. Having high-risk patients adequately prepared when dialysis becomes suddenly needed can mean either a rapid outpatient dialysis start or at least a shorter length of stay in the hospital for dialysis initiation.

Our study has several limitations. First, we ascertained dialysis start by chart review which could lead to misclassification bias but two independent authors did the ascertainment thus minimizing this bias. Secondly, despite attempting to control for many factors that are known to influence the risk of CKD progression, we cannot account for every variable, and there may be some residual confounding. While we were able to capture granular clinical data, our database did not include potential important factors such as delayed patient decision making regarding dialysis and health literacy, nor could we capture the reasons behind starting planned dialysis with a catheter which could potentially increase the risk of an unplanned start.[2] Lastly, our study is limited to a single Canadian academic health centre. Thus, our experience may not be generalizable to other centers with different patient populations, access to health care, and practice patterns. However, our large catchment area and the fact that we represent the only CKD program in that area help strengthen the validity and potential generalizability of the results.

The strengths of our study include the prospective data entry in our database with continuous verification of the accuracy of our database, and accurate adjudication of unplanned starts being performed by two independent physicians, rather than relying on surrogate measures, such as catheters versus fistula or grafts.

In summary, a large proportion of patients with CKD followed in our multi-disciplinary CKD clinic initiated unplanned dialysis. This is consistent with the currently published literature. Our findings reveal that higher BMI and patients with CHF appear to be much more likely to begin dialysis in an unplanned fashion. Further, we recommend that consideration be given to earlier education, more frequent follow up, and earlier dialysis access creation in this subset of the CKD patient population. Efforts to elucidate the causal pathway behind this and to test the effectiveness of our suggested approach are warranted.
